# COVID-19 in Myanmar: Spread, actions and opportunities for peace and stability

**DOI:** 10.7189/jogh.10.020374

**Published:** 2020-12

**Authors:** Myo Minn Oo, Nilar Aye Tun, Xu Lin, Don Eliseo Lucero-Prisno

**Affiliations:** 1 Epidemiology Unit, Prince of Songkla University, Hat Yai, Thailand; 2 Department of Health Policy and Management, University of Public Health, Yangon, Myanmar; 3 Melbourne School of Population and Global Health, University of Melbourne, Melbourne, Australia; 4 Department of Thoracic Surgery, First Affiliated Hospital, College of Medicine, Zhejiang University, Hangzhou, Zhejiang, PR China; 5 Department of Global Health and Development, London School of Hygiene and Tropical Medicine, London, UK; 6 Faculty of Management and Development Studies, University of the Philippines (Open University), Los Baños, Laguna, Philippines

Since the declaration of pandemic on March 11, 2020, COVID-19 has been spreading to almost every country across the world, leading to a rising tally of more than 14 million cases, and half a million deaths [1]. The pandemic which originated in China, slowly made its way to its neighbouring countries including Cambodia, Laos, Thailand, and Myanmar. With a fragile health system exacerbated by long ongoing civil wars and conflicts, Myanmar is especially vulnerable to the spread of COVID-19 because of its 2227-km porous border with China where workers and migrants cross daily; as well as borders shared with Bangladesh, India, and Thailand, all of which reported higher number of cases. With the announcement of Myanmar’s first two laboratory-confirmed cases on March 23, 2020, it ushered its entry to the countdown of the spread. As of August 27, 2020, there were 580 confirmed cases across Myanmar with 6 deaths (male, 67%) and 345 recoveries [2]. In this commentary, we provide a quick analysis of the current state of COVID-19 pandemic in Myanmar and the country’s efforts to address it and its impact.

Myanmar responded early to the impending COVID-19 breakout with the formation of the Inter-Ministerial Working Committee a few days before the World Health Organization (WHO) declared it as a global health emergency. This was followed by the setting up of the National Central Committee to prevent, control and treat COVID-19, chaired by the State Counsellor Aung San Suu Kyi. It aimed at smoothing out and fast-tracking activities at the central level such as case investigation and management, providing community awareness and disseminating updates regarding the pandemic, and securing funding, procurement of essential medicine and equipment in time. Although this may be considered as a timely and bold move by a developing country, concerns were raised with such a centralized command and control approach.

Following the first two cases, strict containment measures were put in place which included travel restrictions, partial lockdowns, closure of major businesses such as factories and shopping malls, quarantining incoming travellers, banning gatherings of five or more people, imposing stay-at-home orders, and curfews in some major cities. Closing borders and enforcing mandatory quarantine, either in a state-sponsored facility or a charity-based one, were also intensified. State Counsellor Aung San Suu Kyi, appeared constantly on television, encouraging the public to bravely face the pandemic and urged them to follow the public health advice provided by the Ministry of Health and Sports (MOHS).

The most remarkable decision by the government was to impose the lockdown during the country’s ten-day long symbolic traditional New Year holidays in April, known as “Thingyan” [3]. Due to lack of strict adherence or poor enforcement of such measure, case numbers almost quadrupled, with majority of cases coming from residents of Yangon, the most populous city in Myanmar. Despite the ban on mass gatherings, a cluster of 50 cases were linked to a religious event, resulting in legal action against a few prominent religious leaders and bifurcation of public opinions amidst the pandemic. These events were further fuelled by an increase in cases during and after a large influx of more than 15 000 Myanmar migrants who returned home by government-sponsored relief flights from Thailand, China, Malaysia and the United Arabs of Emirates. While facility-based or community quarantines were planned in areas with returning migrant workers, some were missed by the system and subsequently overwhelmed the health system which complicated contact tracing, leading to ineffective and inefficient control.

Myanmar’s health sector is already challenged by common health problems faced in developing countries, including shortage of skilled health care workers and underfunded health infrastructure. It has a population of just over 54 million, and a population density of 83 per square kilometre. Myanmar’s health care consistently ranks the world’s lowest and it was included in 57 countries facing critical health workforce shortages. Majority of its States and Regions are well below the WHO recommended minimum number of 1 per 1000 population for medical doctors: with a wide range of disparities in the number of urban to rural doctors. There were 6.7 doctors, and 10 nurses and midwives per 10 000 population in 2018 [4]. In early 2020, there were about 600 critical care beds and 180 ICU beds across the country, which is roughly 1.1 bed per 100 000 people. Testing capacity was initially limited to only two facilities, but later extended to another three facilities including that of the military sector. All these rendered a huge blow on the frail health system and limited the response to COVID-19. The same health system is already struggling to cope with the conventional health demands. Thus, careful consideration should be given to resource re-allocation, alleviating health system exhaustion and upgrading health staff capacity.

The health care workers have been affected heavily by COVID-19 compared to other professions as they are directly responsible for the well-being of patients. Health workers are challenged by issues, such as exhaustion due to heavy workload, inadequacy or lack of personal protective equipment (PPEs) and the fear of getting and/or spreading the infection [5]. In Myanmar, 14 health workers have been infected with COVID-19. The shortage of health staff and the increasing social tension they are exposed to, the increased level of verbal aggression, social stigma, violence and even attacks aggravate the situation. Fortunately, due to the cultural norms and social positioning of health workers within the community, Myanmar has not yet seen such violence during the pandemic.

Though COVID-19 is a public health threat mobilizing the public health sector, politics and humanitarian vulnerabilities are also playing vital roles in responding and acting on the public health priorities. The current centralized “whole-of-government” approach taken by the Myanmar government may likely be a challenge since there are various political players and scenarios that come into play, as well as different degrees of peace, security, non-governmental control, and conflicts across Myanmar [6,7]. Although many ethnic armed groups in Myanmar appear willing to put aside differences and work with the central government to help address the pandemic, conflict is still escalating in northern Rakhine State which is beyond the control of the Myanmar central government. In 2019 alone, 80 000 people have been displaced due to conflict and violence, making the total number of internally displaced populations at 457 000 [6]. Despite the domestic and international calls for a ceasefire, fighting continues in Karen, Shan, Rakhine, and Chin states amidst COVID-19. This situation places health workers at serious risk to health and conflict. The situation at the border with Bangladesh convolutes the risky situation [8]. Hence, the need for a nationwide ceasefire during this pandemic. A tailored approach for each area should be figured out through coordination and cooperation among the government, non-government groups, and humanitarian agencies [9].

**Figure:**
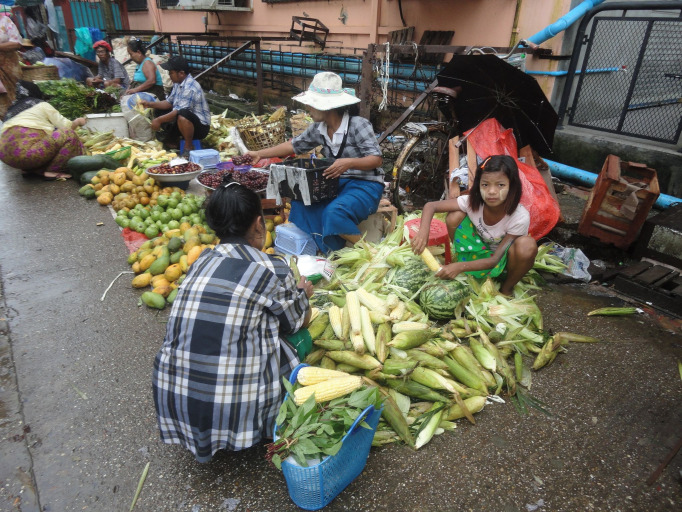
Photo: From the author’s own collection, used with permission.

Civil society organizations (CSO) can play an important role in addressing COVID-19 in Myanmar. A large spectrum of such CSOs are operating across the country, particularly at the grassroots and villages level. They are organised and formalised with linkages to the larger township or regional networks. CSOs take the form of religious or ethnic solidarity groups. Despite their significant number, they have yet to be tapped. They may have limitations in terms of finance, scale of operation, service coverage, and capacity, but they have the advantage of strong community-level engagements. It is proposed that mitigation efforts may use these organizations so as to localize approaches, while the central government provides support and the coordinating role. This community-based approach would be an advantage amidst Internet blackouts and media shutdowns in states like Rakhine. These information outages have detrimental effect on the public in accessing vital public health information. The effects can be lessened by utilizing and coordinating the efforts of existing CSOs inside such areas. Community awareness of public health initiatives, sanitation and hygiene practices and disease prevention strategies can be supported more efficiently and effectively through person-to-person CSO-led communication augmented by the radio [10]. Sustainability of these initiatives should be considered as these are largely influenced by livelihood, economic and social conditions—factors which had negative effects on some COVID-19 measures.

## CONCLUSION

Many countries in South-East Asia, including Myanmar, are still facing a slow-paced pandemic and yet to face its full impact. The current actions of the government of Myanmar are hinged on sustaining the current benign situation, while taking precautionary measures against the further spread, health system exhaustion, public complacency of preventive measures, and livelihood instability and insecurity due to the long-term economic shutdown. Myanmar is performing, so far, seemingly well in its task in tackling COVID-19. The country may use this opportunity to fortify the call for peace across the country and strengthen the resilience of the health system. This situation pushed Myanmar to revisit its investment in health. There is no doubt that the health sector of a nation can be revitalised in times of great adversity. Myanmar joins the world in looking forward to an effective treatment or vaccine. Until that time, all stakeholders in Myanmar need to put aside differences, unify and continue to strengthen its efforts in containing COVID-19.
